# The complete chloroplast genome sequence of *Belosynapsis ciliata* (Blume) R. S. Rao (Commelinaceae)

**DOI:** 10.1080/23802359.2019.1567288

**Published:** 2019-07-12

**Authors:** Wei Guo, Yufen Xu, Wei Wu, Youhua Ye, Ping Chen

**Affiliations:** Department of Horticulture and Landscape Architecture, Zhongkai University of Agriculture and Engineering, Guangzhou, China

**Keywords:** *Belosynapsis*, Commenlinaceae, complete chloroplast genome, automated assembly

## Abstract

*Belosynapsis* is a small genus of mainly perennial herbs in the family Commenlinaceae, comprising about six species native to Southeast Asia, the Indian subcontinent, Papuasia, and southern China. An ideal alternative plant for groundcover or rooftop greenery, the lack of genetic resources have impeded further studies on *Belosynapsis ciliata* and thus its utilization. In this study, we report the complete chloroplast genome of *B. ciliata*, assembled from Illumina paired-end genome resequencing data. The assembled chloroplast genome was 170,870 bp in length, comprising a large single-copy (LSC) region of 97,374 bp, a small single-copy (SSC) region of 20,224 bp, separated by two inverted repeat (IR) regions of 26,636 bp each. A total of 132 genes were predicted with an overall GC content of 34.53%. *Belosynapsis ciliata*, belonging to the order Commelinales, was well separated from species of other orders of Arecales, Poales, and Zingiberales, with high support.

*Belosynapsis* is a small genus in the family Commenlinaceae, comprising about six perennial species distributed in the Southeast Asia, the Indian subcontinent, Papuasia, and southern China (Hong and DeFilipps 2000). The species *Belosynapsis ciliata* is a creeping herb with purple or pink capitulum and its somewhat succulent leaf confers it extraordinary drought tolerance. Hence, this species is an ideal alternative plant for groundcover or rooftop greenery in tropical or subtropical areas. As the first step to promote further study and utilization of this species, we report the assembly of the complete chloroplast genome sequence of *B. ciliata*, the first for the family Commenlinaceae so far.

We sampled an individual of *B. ciliata* from Huadu, Guangzhou, China, and deposited a voucher specimen (BEL-2018-GZ01) at the Sun Yat-Sen University Herbarium (SYS). Fresh leaf material was used for the isolation of total genomic DNA and a library was constructed prior to sequencing on an Illumina **HiSeq** 2500 System (San Diego, California, U.S.) (paired-end 250 bp). Using a seed-and-extend algorithm implemented in NOVOPlasty (Dierckxsens et al. 2017), we assembled the chloroplast genome of *B. ciliata* using approximately 4 Gb of data as input and the *rbc*L gene sequence of *B. ciliata* (GenBank accession HQ182418.1) as seed. After the assembly, we randomly chose and Sanger-sequenced seven genes on the genome to verify the accuracy of the automated assembly. DNA sequences of the seven genes (*rps*16, *atp*F, *rpo*C2, *rpo*C1, *atp*E, *mat*K, and *ndh*D), covering approximately 10,479 bp (6.1% of the total chloroplast genome size) had 100% match to those on the assembled genome. Using Verdant (McKain et al. 2017) and DOGMA (Wyman et al. 2004), the chloroplast genome was annotated with manual corrections.

The length of the complete chloroplast genome sequence of *B. ciliata* (GenBank accession MK133255) was 170,870 bp, with a large single-copy (LSC) region of 97,374 bp, a small single-copy (SSC) region of 20,224 bp, separated by two inverted repeat (IR) regions of 26,636 bp each. A total of 132 genes were predicted, including 87 protein-coding genes, 37 tRNA genes, and 8 rRNA genes. The overall GC content was 34.53%. The *pet*D gene had an AUA start codon, instead of the conventional AUG start codon.

For phylogenetic tree construction, the chloroplast genomes of *B. ciliata* as well as 14 other commenlinids and one outgroup species *Calanthe triplicata* (NC_024544.1) downloaded from GenBank, were aligned using MAFFT v7.307 (Katoh and Standley 2013). A maximum likelihood tree (Figure 1) was then constructed using RAxML (Stamatakis 2014). *Belosynapsis ciliata* in the order Commelinales was well separated from the other orders, including Arecales, Poales, and Zingiberales, with high support.

**Figure 1.**
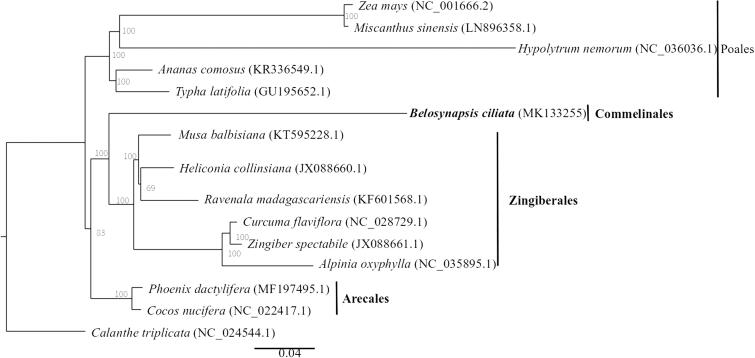
Maximum likelihood tree of Commenlinids based on complete chloroplast genomes, with *Calanthe triplicata* as outgroup. Bootstrap support values (based on 1000 replicates) are shown next to the nodes. Scale in substitutions per site.
